# Supplementary material for: The Flagellar Transcriptional Regulator FtcR Controls Brucella melitensis 16M Biofilm Formation via a betI-Mediated Pathway in Response to Hyperosmotic Stress
Source: Int J Mol Sci. 2022 Aug 31;23(17):9905. doi: 10.3390/ijms23179905 (PMC9456535; doi:10.3390/ijms23179905)
Supplement: Supplementary file 1 [file ijms-23-09905-s001.zip › ijms-1881473-Supplementary Material.pdf]

Supplementary material

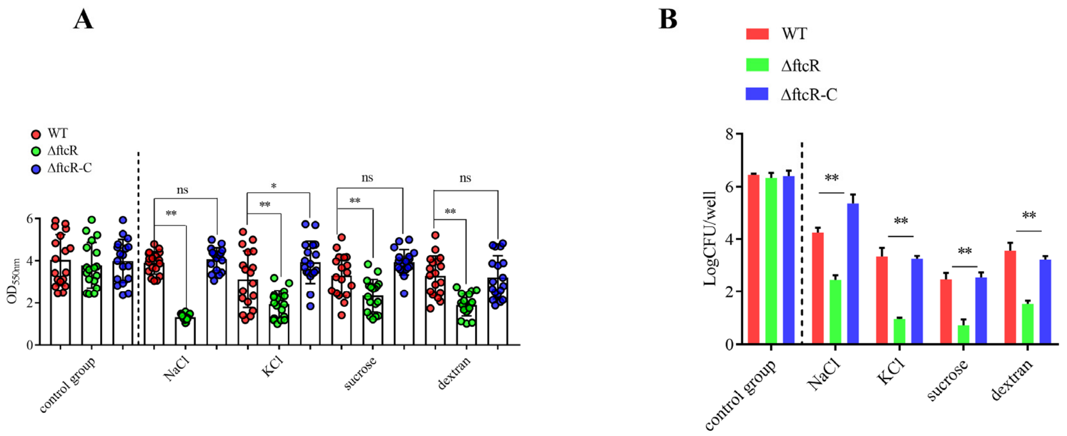

Supplementary Figure S1. Biofilm resistance of Δ*ftcR* in response to different osmotic solutes. (**A**) The biofilms of WT, Δ*ftcR*, and Δ*ftcR*-C were treated with different osmotic solutes for 20 d and their biomass was quantified using a crystal violet (CV) assay. Each point represents an independent well. (**B**) Survival of WT and Δ*ftcR* cells in biofilms grown under different osmotic solutes. Error bars represent standard error (n ≥ 3). * $p \leq 0.05$, ***$p \leq 0.01$, unpaired Student's *t*-test. ns, not significant.

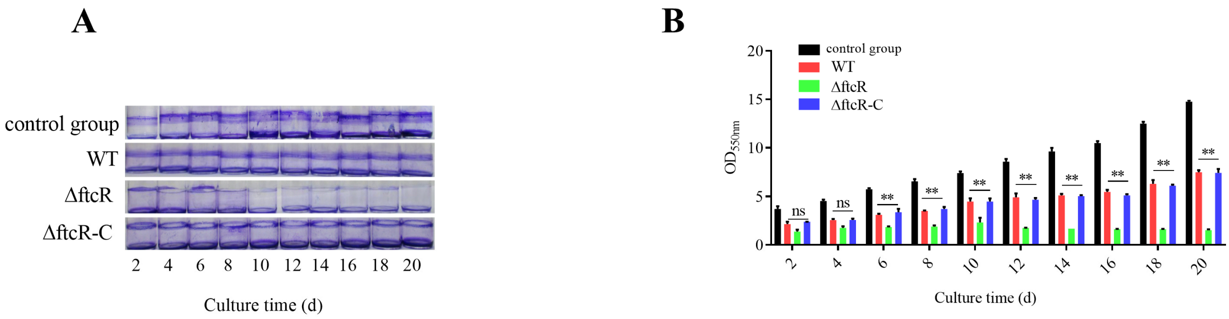

Supplementary Figure S2. Quantitative analysis of the biofilm using CV staining and bacterial plate count. (**A**) The biofilm of the WT, Δ*ftcR*, and Δ*ftcR*-C strains under hyperosmotic stress and quantified with 0.1% CV. (**B**) Survival of WT, Δ*ftcR*, and Δ*ftcR*-C strains in biofilms grown under hyperosmotic stress. Error bars represent standard error (n ≥ 3). **$p \leq 0.01$, unpaired Student's *t*-test. ns, not significant.

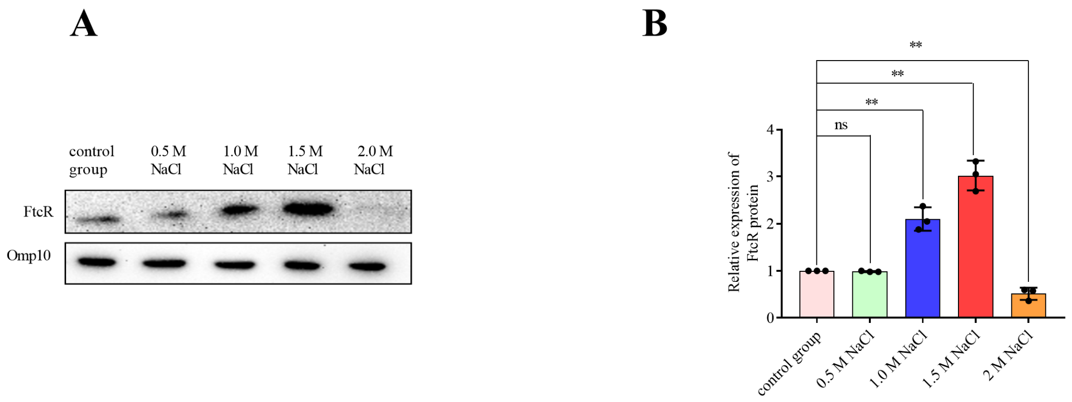

Supplementary Figure S3. FtcR protein expression in biofilm under hyperosmotic stress conditions. (**A**) Expression of FtcR in biofilm cells after NaCl treatment was detected by western blotting. (**B**) Protein expression was evaluated by measuring the mean gray values of western blots using ImageJ. Error bars represent standard error (n ≥ 3). **$p \leq 0.01$, unpaired Student's *t*-test. ns, not significant.

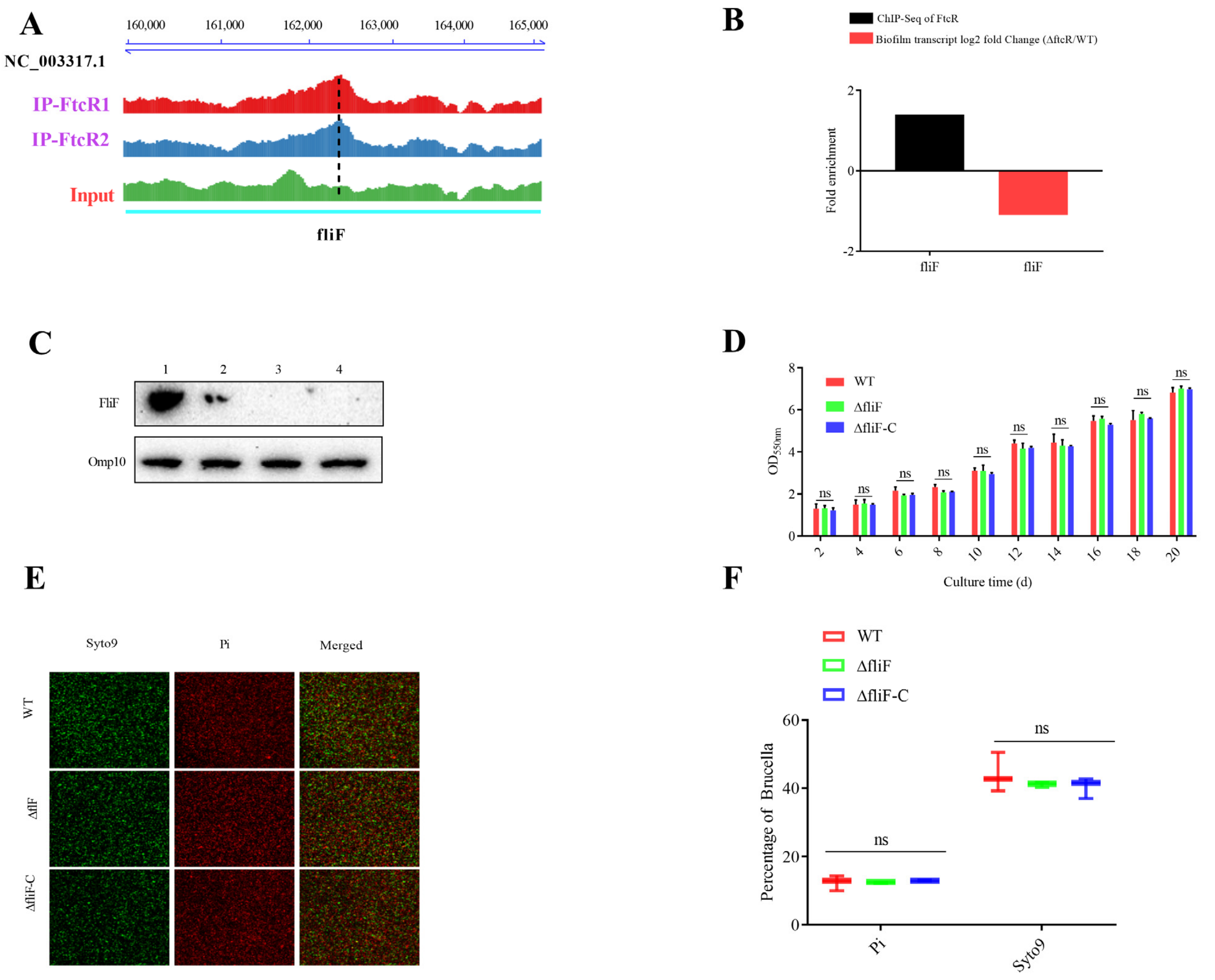

Figure S4. Effects of FliF on biofilm formation. (**A**) FtcR-enriched sequence reads located within the promoter regions of *fliF*, with annotated genes shown at the bottom—ChIP1 and ChIP2—from two independent experimental replicates. Scale bar: 1 kb. (**B**) The fold enrichment analysis of *fliF* in RNA-Seq and ChIP-Seq. (**C**) Expression of FliF distributed in logarithmic, biofilm matrix, cell of the early logarithmic growth (line 1), late logarithmic growth (line 2), biofilm under control condition (line 3), and biofilm under hyperosmotic stress (line 4) using western blot. (**D**) Biofilm growth of WT, Δ*fliF* and Δ*fliF*-C cultures was monitored in the presence of the 1.5 M NaCl for 20 d and the biomass was quantified with 0.1% CV. (**E**) Live confocal imaging of biofilms formed by WT, Δ*fliF*, and Δ*fliF*-C cells labeled using Syto9 and PI fluorescent probes. (**F**) Flow cytometry analysis of the live/dead status of bacteria in the biofilm filtrates. Error bars represent standard error (n ≥ 3). Unpaired Student's *t*-test. ns, not significant.

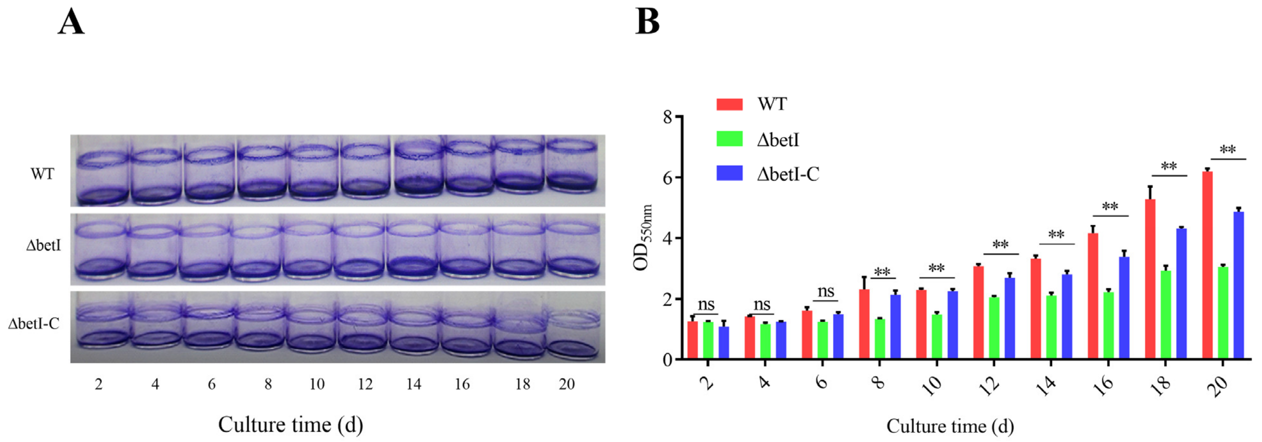

Figure S5. Quantitative analysis of the biofilm using CV staining and bacterial plate count. (**A**) The biofilm of WT, Δ*betI*, and Δ*betI*-C strains under hyperosmotic stress and quantified with 0.1% CV. (**B**) Survival of WT, Δ*betI*, and Δ*betI*-C strains in biofilms grown under hyperosmotic stress. Error bars represent standard error (n ≥ 3). $^{**}p \leq 0.01$, unpaired Student's *t*-test. ns, not significant.

Table S1 FtcR binding sites identified by ChIP-Seq and RNA-Seq.

Chromosom[a]	Peak start (bp)	Peak stopa (bp)	ChIP fold enrichment (IP/Input)	Downstream gene[b]			Biofilm transcript log2 fold Change (Δ*ftcR*/WT)
				ID[c]	Name	Product annotation	
				**Transport and binding proteins**			
NC_003317.1	159,616	162,822	20.41	BME_RS00790	amtB	ammonium transporter	-1.95
NC_003317.1	1,431,703	1,432,149	4.74	BME_RS06945	betI	transcriptional regulator BetI	-2.29
NC_003317.1	165,608	169,298	1.70	BME_RS00805	BME_RS00805	DMT family transporter	-1.3
NC_003317.1	1,462,803	1,463,632	1.48	BME_RS07095	BME_RS07095	ABC transporter permease	1.82
NC_003317.1	1,405,154	1,405,417	1.45	BME_RS06800	BME_RS06800	penicillin-binding protein 1A	-1.52
NC_003317.1	1,418,321	1,418,648	1.37	BME_RS06870	BME_RS06870	S9 family peptidase	1.91
NC_003317.1	1,989,144	1,989,754	1.40	BME_RS09570	BME_RS09570	extracellular solute-binding protein	1.19
NC_003317.1	466,058	466,440	1.32	BME_RS02235	BME_RS02235	Lrp/AsnC ligand binding domain-containing protein	1.41
NC_003318.1	950,346	950,574	1.30	BME_RS14635	BME_RS14635	amino acid permease	1.97
NC_003317.1	1,122,809	1,123,018	1.14	BME_RS05400	BME_RS05400	ATP-binding protein	1.26
NC_003318.1	563,019	563,278	1.27	BME_RS12810	BME_RS12810	ABC transporter ATP-binding protein	-1.15
NC_003317.1	1,598,343	1,598,611	1.25	BME_RS07740	sbmA	peptide antibiotic transporter SbmA	1.18
NC_003317.1	278,153	278,501	1.21	BME_RS01300	BME_RS01300	MFS transporter	1.11
Regulatory functions							
NC_003317.1	170,084	170,669	3.17	BME_RS10945	ftcR	response regulator transcription factor	-1.80
NC_003317.1	2,030,864	2,031,583	1.53	BME_RS09760	BME_RS09760	helix-turn-helix domain-containing protein	1.13
NC_003317.1	1,275,020	1,275,763	1.52	BME_RS06170	BME_RS06170	helix-turn-helix domain-containing protein	1.13
NC_003318.1	840,458	840,828	1.29	BME_RS14130	BME_RS14130	CerR family C-terminal domain-containing protein	-2.09
NC_003318.1	676,928	677,262	1.25	BME_RS13320	BME_RS13320	IclR family transcriptional regulator	-1.58
NC_003318.1	439,149	439,520	1.38	BME_RS12245	BME_RS12245	IMPACT family protein	-1.22
NC_003317.1	1,731,455	1,731,741	1.21	BME_RS08395	BME_RS08395	SANT/Myb-like DNA-binding domain-containing protein	-1.07
				Cell envelope			

NC_003317.1	976,229	977,214	1.80	BME_RS04695	mscS	Small-conductance mechanosensitive channel	1.45
NC_003318.1	161,916	162,299	1.42	BME_RS10900	BME_RS10900	autotransporter outer membrane beta-barrel domain-containing protein	-1.32
NC_003317.1	609,170	609,467	1.30	BME_RS02920	bamD	outer membrane protein assembly factor	1.21
Translation							
NC_003317.1	1235	2280	1.63	BME_RS00020	rhO	transcription termination factor	1.38
NC_003317.1	796,452	796,668	1.25	BME_RS03875	rplO	50s ribosomal protein L15	1.61
Hypothetical proteins							
NC_003317.1	562,645	562,929	1.27	BME_RS02710	BME_RS02710	hypothetical protein	1.35
NC_003317.1	1,464,965	1,465,199	1.25	BME_RS07105	BME_RS07105	hypothetical protein	1.57
NC_003317.1	736,280	736,631	1.62	BME_RS03530	BME_RS03530	hypothetical protein	-1.39
NC_003317.1	1,014,836	1,015,571	1.29	BME_RS04850	BME_RS04850	DUF2059 domain-containing protein	1.40
NC_003317.1	1,263,340	1,263,620	1.50	BME_RS06115	BME_RS06115	SH3 domain-containing protein	-1.07
NC_003318.1	309,516	309,817	1.39	BME_RS11645	BME_RS11645	hypothetical protein	-1.23
NC_003317.1	540,668	540,910	1.36	BME_RS16045	BME_RS16045	hypothetical protein	2.08
NC_003317.1	1,359,006	1,359,221	1.23	BME_RS06590	BME_RS06590	DUF423 domain-containing protein	-2.08
NC_003318.1	782,398	782,682	1.31	BME_RS03750	BME_RS03750	hypothetical protein	1.36
Fatty acid and phospholipid metabolism							
NC_003317.1	929,833	930,378	1.56	BME_RS04465	BME_RS04465	acyl-CoA dehydrogenase	1.17
NC_003318.1	849,100	849,440	1.52	BME_RS14185	BME_RS14185	acyl-CoA synthetase	-1.26
NC_003317.1	826,002	826,710	1.29	BME_RS03995	BME_RS03995	acyl-CoA carboxylase subunit beta	1.02
NC_003318.1	126,309	126,561	1.24	BME_RS10785	BME_RS10785	amidohydrolase	-1.62
Cellular processes							
NC_003317.1	1,121,712	1,122,020	1.29	BME_RS05390	secDF	protein translocase subunit SecDF	1.36
NC_003318.1	363,874	364,095	1.26	BME_RS11895	BME_RS11895	D-aminopeptidase	-1.8
NC_003317.1	521,102	521,437	1.44	BME_RS02510	BME_RS02510	lytic transglycosylase domain-containing protein	-1.13
NC_003317.1	1,034,504	1,034,944	1.30	BME_RS04940	BME_RS04940	septal ring lytic transglycosylase RlpA family protein	1.15
NC_003317.1	1,093,915	1,094,349	1.32	BME_RS05270	prfB	peptide chain release factor 2	1.80
NC_003318.1	29,861	30,171	1.24	BME_RS10340	BME_RS10340	type IV secretion system protein	-2.51

NC_003317.1	2,033,625	2,033,923	1.23	BME_RS09770	BME_RS09770	hemolysin family protein	1.17
NC_003317.1	1,674,958	1,675,197	1.25	BME_RS08095	BME_RS08095	glycosyltransferase	-1.27
NC_003317.1	1,462,130	1,462,520	1.25	BME_RS07090	BME_RS07090	DegT/DnrJ/EryC1/StrS aminotransferase family protein	1.78
NC_003317.1	1,009,588	1,010,451	1.24	BME_RS04825	gltX	glutamate-tRNA ligase	1.06
NC_003317.1	1,027,230	1,027,614	1.23	BME_RS04910	BME_RS04910	MBL fold metallo-hydrolase	1.30
NC_003318.1	716,427	716,639	1.31	BME_RS13530	BME_RS13530	BA14K family protein	-1.09
NC_003317.1	555,294	557,788	1.82	BME_RS02665	BME_RS02665	YcgN family cysteine cluster protein	1.49
NC_003317.1	1,345,304	1,345,620	1.43	BME_RS06525	BME_RS06525	NYN domain-containing protein	1.28
Energy metabolism							
NC_003317.1	1,451,282	1,451,487	1.14	BME_RS07020	BME_RS07020	mannose-1-phosphate guanylyltransferase/mannose-6-phosphate isomerase	1.26
NC_003318.1	367,936	368,158	1.29	BME_RS11925	BME_RS11925	Gfo/Idh/MocA family oxidoreductase	-1.62

[a]ChIP-Seq peaks of FtcR distributed across the *Brucella melitensis* 16M genome in chromosomes I and II.

[b]Lists the gene located downstream from the target site and whether the gene is changed in the transcriptome.

[c]ID, identifier.

Table S2. Primers of mutants and complementation used for this work.

Primer	Sequence
ftcR-N-F	GCCGAGCCTGAATGTGGAC
ftcR-N-R	GACATTCATCCCAGGTGGCCGGCCCCGCCTTCC
ftcR-C-F	TCTGGGGTTCGAAATGACCGTATTGATGTTTTCCCGCAAGTC
ftcR-C-R	GGGCAGGACAAGCGAGG
kan-F	GCCACCTGGGATGAATGTC
kan-R	CGGTCATTTCGAACCCCAGA
fliF-N-F	CTGACACTGATGCGAAGA
fliF-N-R	GACATTCATCCCAGGTGGCCCTGTTGAACGCAAGCTA
fliF-C-F	TCTGGGGTTCGAAATGACCGGAATGAGCGCGCTTT
fliF-C-R	ATGGGCCTGCAATGC
betI-N-F	CTTTGGGCAAGCTTTACC
betI-N-R	GACATTCATCCCAGGTGGCGATATTCATCCGGTTGTCTG
betI-C-F	TCTGGGGTTCGAAATGACCGGCATGGAAGCAGATTTCG
betI-C-R	GGCATGGAACAGCGGATTG
ftcR-F-C	AAGCTTGGATGATTGTTGTCGT
ftcR-R-C	GAATTCGGTCACTCGATATTGA
betI-F-C	CGTCGACTGCCCAAGATCGGGA
betI-R-C	TGAATTCTCAATTCTCCGCCGAA
p2c2Xamont	ATATCTAGATTCAGCCGCGGCGGGCT
p2c2Baval	ATTGGATCCCCTTCGCGACCGAACCA
Biotin-ftcR-F	GACGGCAGCGGGCAAAAGCC
Biotin-ftcR-R	TTCTGCTGCCGCCTTATCAA
Mut-ftcR-F	GACGGCTGTCCTTGAAAGCC
Mut-ftcR-R	TTCTGCTGCCGCCTTATCAA
Biotin-betI-F	TGCCTGCCGCTTTGGGCAAG
Biotin-betI-R	TGTTTTTTCGCTGGGCGACATAGAA
Mut-betI-F	TGCCTGCCGCTGTCCTTGAG
Mut-betI-R	TGTTTTTTCGCTGGGCGACATAGAA

Table S3. Primers of qRT-PCR used for this work.

Primer	Sequence
16S rRNA-F	ACTAAGGGCGAGGGTTGC
16S rRNA-R	CACTGGACCATTACTGACGC
amtB-F	AGCCTGCCCGCACTTT
amtB-R	TCTTGCGCACCATGCC
betI-F	TGCCATCCGCACCATC
betI-R	GTTTGATCGCCGCGTT
BME_RS00805-F	GGGTGCTGATCTTTTCCGGG
BME_RS00805-R	CGACAACCAGTGAAACAGCA
BME_RS07095-F	TGGAAGGTACGCCACTTC
BME_RS07095-R	ATATAGGGGGCATATGCA
BME_RS06800-F	GGCAGAAAAAGATCGCGG
BME_RS06800-R	GCCGGGAGAAAATCGTGA
BME_RS06870-F	ACCATGGCCGACCTTGAA
BME_RS06870-R	CCCACACCGAGGAAAAAG
BME_RS09570-F	GGTGGCCATGCTTGCAAG
BME_RS09570-R	TTGAGGTTGTCGAAAGTGCC
BME_RS02235-F	TCGCAGACGGCCACC
BME_RS02235-R	GGCGTCGTGCGGTCA
BME_RS14635-F	ATGACAACACCAACCGCA
BME_RS14635-R	GAAAGAAAACGGCTGCAA
BME_RS05400-F	TGGACCGTTTGATAGCCG
BME_RS05400-R	CCAAATCTACGCCACGGA
BME_RS12810-F	CCTGCATGATGTCTCGCT
BME_RS12810-R	GCATTGCCCAGAAGCACT
sbmA-F	CCCCGTCCGAAACTG
sbmA-R	GAACACATGCGCGCC
BME_RS01300-F	GATATGGCGCCTGCG
BME_RS01300-R	AGCGAACCGAGACCG
